# Strategic ownership configurations to foster intangible cultural heritage in Japanese football events

**DOI:** 10.3389/fspor.2025.1638764

**Published:** 2025-08-18

**Authors:** Ricardo Gúdel, Emilio Hernández-Correa, Fernando Acebes, Javier Pajares

**Affiliations:** Department of Business Organization and Marketing, University of Valladolid, Valladolid, Spain

**Keywords:** ownership landscape (OL), resource-based view (RBV), intangible cultural heritage (ICH), sports governance, Japanese football, bottom-up management, sports event, community engagement

## Abstract

**Introduction:**

This study examines how different configurations of the Ownership Landscape influence the preservation of Intangible Cultural Heritage in sporting events, using Japan as a case study. Drawing on the Resource-Based View, it explores how ownership models act as strategic resources that affect both cultural sustainability and long-term competitiveness.

**Methods:**

A qualitative multiple-case study was conducted on four football-related events: the J.League, the All Japan University Football Championship, the Shakaijin Cup, and local festival-based tournaments. The study combined documentary analysis with Data Envelopment Analysis to assess how governance and ownership structures generate valuable, rare, inimitable, and non-substitutable resources.

**Results:**

Findings reveal that participatory and hybrid ownership models are more effective in activating VRIN resources, ensuring both organizational resilience and cultural embeddedness. A blend of national sponsorship and local identity in professional leagues, the academic traditions of the Intercollege, and the Shakaijin Cup's regional roots show how sporting events reflect and generate cultural forms.

**Discussion:**

This research challenges top-down and purely economic evaluations of sports events, advocating for culturally sensitive governance that fosters local engagement and authenticity. It extends the RBV to multi-actor, culturally embedded ecosystems and proposes adaptable principles: promoting hybrid ownership, embedding cultural narratives, and engaging diverse stakeholders. Sporting events, when aligned with community values, serve as platforms for intergenerational cultural transmission and community development.

## 1 Introduction

Sports events are not merely arenas of competition or entertainment; they increasingly serve as dynamic cultural institutions deeply embedded within community identities (1, 2). As recognized by UNESCO (3), local sports practices make a significant contribution to the preservation and intergenerational transmission of intangible cultural heritage (ICH). Through recurring events, communities create and perpetuate symbols, rituals, and narratives that reinforce collective memory and shape local identities (4, 5). Thus, sports competitions become critical sites for performing, revitalizing, and sustaining cultural traditions and values.

Japan exemplifies a unique integration of sport and cultural heritage as they are rooted in longstanding practices of preserving intangible traditions and community values. For instance, traditional games such as kemari have been officially designated as cultural properties (6). Concurrently, modern sports, notably football, since the establishment of the J.League in 1993, have adopted hybrid governance models that merge corporate management with local community involvement (7). Japanese football clubs exemplify how sports can promote strong local identities and cultural expressions within a framework that integrates traditional values within modern organisational strategies (8, 9).

However, despite growing scholarly interest in sports heritage, a notable research gap remains regarding how event-level governance and management structures influence the cultural significance of sporting events. Current literature often compartmentalises sport events studies into economic and marketing impacts (10, 11), soft power (12), tourism (2, 13) or purely heritage preservation contexts (14), thus neglecting their intersection. Specifically, the interaction between top-down and bottom-up management approaches in shaping the cultural relevance of sporting events is inadequately studied. This is especially relevant in culturally hybrid settings such as Japan.

Addressing this gap is crucial for enriching sports management theoretical frameworks and practical governance and policy implications. Applying the Resource-Based View (RBV) (15, 16), this article utilizes the Ownership Landscape (OL) (17) as a novel conceptual tool for analyzing how the strategic distribution and interaction of stakeholders influence the cultural and organizational dynamics of sports events. Thus, by examining OL through the RBV lens, this study addresses the following research question: how specific resource configurations in the governance and management of sporting events can promote enduring cultural legacies?

Accordingly, the main objective of this research is to explore the relationship between bottom-up governance strategies in Japanese football events and their respective OLs, evaluating impacts on ICH preservation and local talent development. Specifically, the study seeks to define and operationalise the OL within the RBV framework, analyse the interaction between top-down and bottom-up governance strategies in the sustainability of Japanese sports events, and assess how diverse OL configurations facilitate the preservation of ICH.

To address these analytical dimensions, the study employs a qualitative-descriptive approach, based on multiple case studies and an extensive review of specialized academic literature. First, the research defines and operationalises the concept of OL as a strategic intangible resource, applying the RBV framework to sports events. Second, it examines how the interaction between bottom-up and top-down governance in the different OL configurations shapes Japan's cultural relevance and organisational sustainability of football competitions. Third, it examines how specific OL configurations contribute to preserving Intangible Cultural Heritage (ICH) and developing grassroots sporting talent. The paper includes an efficiency assessment of J1, J2 and J3 clubs using DEA (Data Envelopment Analysis) indicators to complement the study of how OL configurations translate into sustainability outcomes. The analysis draws on four emblematic cases in Japanese football: the Japanese professional football leagues (J1, J2 and J3 League), the All Japan University Football Championship, the Shakaijin Cup, and diverse football initiatives embedded within traditional local festivals.

The paper is structured as follows. First, it presents the theoretical framework, outlining the RBV and the concept of OL as applied to sports event management. Next, the methodology describes the qualitative-descriptive case study approach, spotlighting four representative cases in Japanese football: the J.League, the All Japan University Football Championship, the Shakaijin Cup, and various local festivals. The subsequent results section analyses governance models and OL configurations across these events. The discussion section interprets these findings concerning previous literature and their theoretical and practical implications. Finally, the conclusion summarizes the key contributions and suggests future research directions.

## 2 Theoretical framework

Sports events extend beyond their commercial or entertainment functions to become living cultural manifestations embedded within communities' collective memories (18). They are moments where rituals, symbols, and practices are performed, renewed, and transmitted across generations, fulfilling a vital role in preserving and dynamising ICH. In line with the UNESCO Convention for the Safeguarding of the Intangible Cultural Heritage (101), ICH is understood as “the practices, representations, expressions, knowledge, skills, as well as the instruments, objects, artefacts and cultural spaces associated therewith, that communities, groups and, in some cases, individuals recognize as part of their cultural heritage” (Article 2).

At the same time, from an organisational standpoint, sports events are complex systems of resource mobilisation, requiring the coordination of tangible assets, such as infrastructure, financial capital, and human resources. Likewise, sport events require intangible assets, such as brand reputation, community loyalty, and cultural identity (19). Thus, understanding sports events as dual carriers of cultural and organizational resources provides a richer analytical lens to study their long-term sustainability and strategic significance.

The Resource-Based View (RBV) was developed as a framework to understand how firms achieve and maintain sustained competitive advantages through the possession and strategic deployment of resources that are valuable, rare, imperfectly imitable, and non-substitutable (VRIN) (15, 16). In this theory, resources encompass physical, human, and organisational assets that allow organisations to conceive and implement effective strategies. Not all resources contribute equally to competitive advantage; only those fulfilling the VRIN criteria can underpin long-term strategic success and develop dynamic capabilities (20).

Viewing sports events through the RBV lens enables us to better understand why some thrive and evolve, while others gradually decline. It is not just about budgets or stadiums; tangible resources like venues, sponsorship deals, and skilled staff indeed count (21, 22). The intangible assets often make the real difference: the experience built over the years, the reputation a tournament earns, the trust and enthusiasm of the local community, and the cultural rituals that grow around the event (19). These less visible elements can be as critical as financial backing in determining whether an event becomes a lasting part of the social fabric, or just a passing occurrence. Notably, intangible resources are typically more critical for sustaining events over time, as they are harder for competitors to replicate (23) and often constitute the core identity and legitimacy of the event within its socio-cultural environment (24, 25).

Building upon the RBV, this study conceptualises OL as a strategic intangible resource. OL refers to the distribution, diversity, and interaction of stakeholders who possess, control, or contribute critical resources to the event (17). These stakeholders include corporations, local governments, federations, community groups, and individual supporters. Their collective capabilities, connections, and strategic orientations configure the landscape where resources are mobilised, protected, and developed.

In previous research, OL has been conceptualised as the composition of owners of participant clubs in national football leagues, impacting competition dynamics and league sustainability. However, in this study, we adapt the OL concept to the context of sports events, where it refers broadly to the distribution and configuration of key stakeholders who possess, control, or contribute with strategic resources necessary to the event's survival and its cultural relevance. While the underlying logic remains consistent, viewing OL as a strategic intangible resource, the focus shifts from club ownership to event governance and resource mobilisation architecture.

Under certain conditions, the OL can meet the well-known VRIN criteria, which help explain why some models become enduring and strategically effective (see Table 1). An OL is valuable when it enables strategies that boost performance and foster long-term sustainability and cultural relevance. It becomes rare when it reflects a distinctive blend of stakeholders and local cultural ties that other events cannot reproduce. Its inimitability lies in the deep historical, relational, and cultural layers that shape it, elements that cannot be easily copied or fast-tracked. It is non-substitutable because alternative setups cannot easily replace the connection between those involved and the specific cultural resources they draw on (26). In short, a well-rooted OL can be much more than an administrative model as it becomes part of what gives an event its essence.

**Table 1:** Summary of the evolution and adaptation of the ownership landscape (OL) concept.

Aspect	Original OL Concept (Leagues)	Adapted OL Concept (Events)
Unit of analysis	National football leagues (CSL, J.League)	Sports events (tournaments, championships, festivals)
focus of OL	Ownership configuration of participant clubs	Ownership and resource configuration of event organizers and stakeholders
Primary actors	Club owners (corporations, investors, state actors)	Event promoters, federations, local governments, community organizations
Strategic role	Shaping competitive balance, identity, and sustainability of the league	Shaping organizational sustainability, community engagement, and cultural heritage of the event
Core RBV logic	OL as a VRIN intangible resource for league advantage	OL as a VRIN intangible resource for event endurance and cultural transmission
Continuity	The underlying premise remains: OL captures the architecture of critical resources that sustain and differentiate organizations/events over time	

Source: own elaboration.

**Figure 1:**
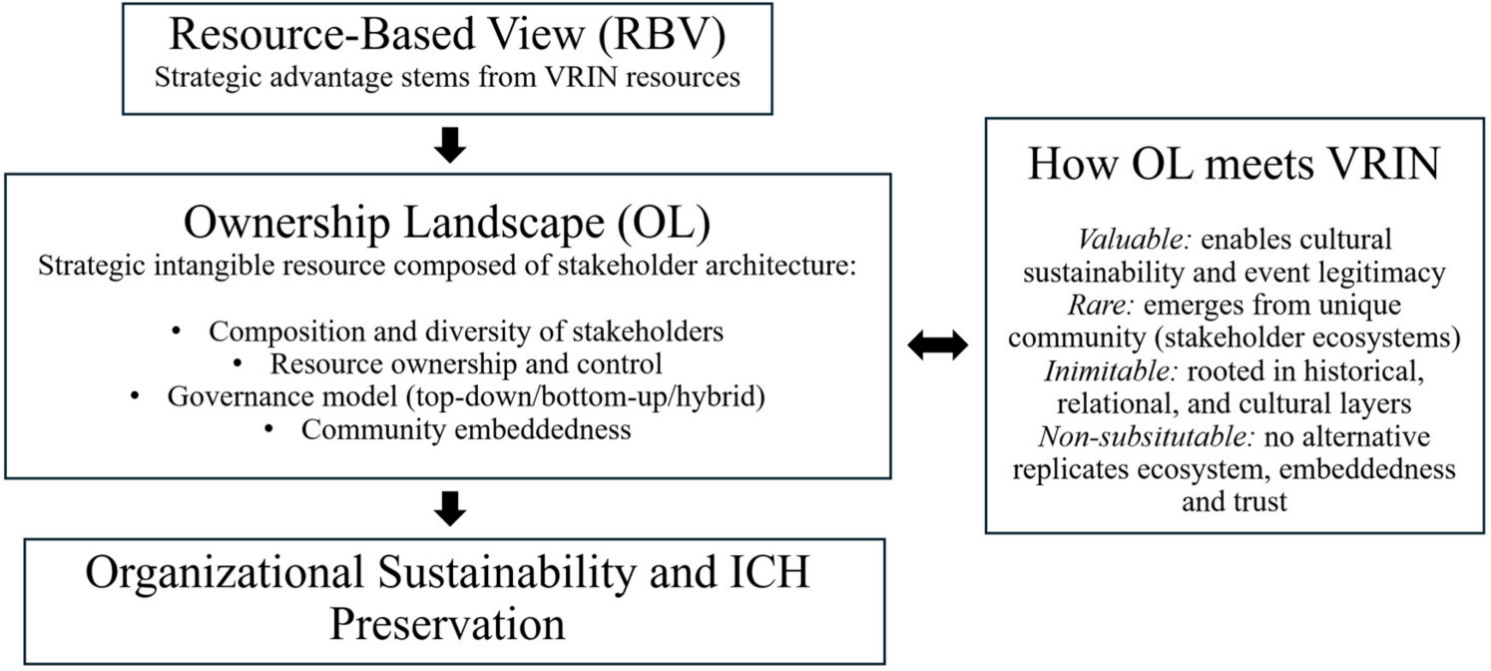
Conceptual integration of the ownership landscape (OL) as a strategic intangible resource within the resource-based view (RBV). Source: own elaboration.

Although prior research has examined the role of stakeholders and relational resources in sport events (19, 27), this study conceptualises OL (Figure 1) as the overall architecture of resource ownership and control among the participants and organisers of an event. In contrast to isolated relational resources, OL refers to the configuration and interplay of stakeholders as a strategic intangible resource that underpins the event's long-term sustainability and capacity for cultural heritage creation. Thus, OL is a systemic resource whose composition, diversity, and governance shape the event's outcomes through a RBV perspective beyond the merely sum of individual relationships.

To differentiate, while OL refers to the configuration of who holds, controls, or contributes key resources to the event, researchers describe governance as the structure and processes through which decisions are made, and strategic directions are implemented (28, 29). Thus, OL is concerned with resource architecture, whereas governance is concerned with management dynamics. Understanding this distinction is crucial as different governance models (top-down, bottom-up, or hybrid) influence how the OL evolves and how resources are mobilised to sustain the event organizationally and culturally (30, 31).

Governance structures critically shape the types of resources that a sports event can develop and sustain (32). In RBV terms, governance acts as a resource development mechanism, determining whether the event fosters tangible assets or intangible assets. First, top-down governance centralises decision-making within a single entity or a small group of elite stakeholders, such as national federations, corporate sponsors, or governmental bodies (33). This model offers advantages in efficiency, resource mobilisation, and strategic coherence. However, it can also limit the organic development of cultural resources if it reduces the participation of grassroots stakeholders and potentially alienates local communities (34). Thus, top-down models can diminish the event's potential to embed itself within the community's cultural heritage (35).

On the other hand, bottom-up governance fosters a more decentralised participatory involvement from local actors such as community organisations, amateur clubs, volunteers, and regional governments (36). In this sense, this type of strategy can do much more than support the logistics of an event, and it can help build real social capital, foster genuine cultural practices, and strengthen resilient forms of organisational knowledge. All these elements contribute to VRIN resources that make an event project. Still, this approach is not without its challenges. Without a clear, centralised strategy, efforts can become fragmented, resources may be spread too thin, and it can be challenging to scale the event or boost its visibility beyond the local level (37).

Hybrid governance models that balance top-down strategic direction with bottom-up community engagement can offer a superior framework for organizationally and culturally sustaining sports events (32, 38). The Japanese experience, particularly within football, illustrates how events can leverage corporate resources while fostering deep-rooted local identities and traditions (39, 40). Such hybrid OLs are conducive to producing events that function as sporting competitions and dynamic platforms for creating ICH and transmission.

In this study, we adopt a grounded approach to ICH, considering it as a set of community-rooted practices, values, and narratives embodied in sport events. These include local rituals, affective fan traditions, symbolic performances (chants, banners, ceremonies), and collective memory linked to clubs and venues. Rather than relying on official listings or heritage registers, we interpret ICH through observed patterns of cultural embeddedness and community participation in football events. This approach aligns with recent scholarship advocating for a performative and relational understanding of ICH in sport and urban contexts (41, 42).

## 3 Materials and methods

This research adopts a qualitative-descriptive approach based on case studies and an extensive review of specialised academic literature. The selection of the Japanese case responds to its unique historical and structural evolution and its cultural significance in sport. Football in Japan provides an exceptional setting for analyzing the intersection between sport and ICH, given its rapid transformation from a traditional corporate structure to more hybrid models that combine community-based and corporate elements (39, 43).

We selected four representative cases within Japanese football to explore this topic in depth, covering different competitive levels and event types. The selection followed a purposive sampling strategy, aimed at capturing the diversity of ownership configurations (OLs) and levels of institutionalisation. These cases were chosen for their empirical relevance and their capacity to represent maximum variation in governance models (bottom-up, top-down and hybrid) and actor configurations for the preservation of ICH. First, we chose the professional leagues as institutionalised events with nationwide reach. Second, the All Japan University Football Championship was included to exemplify the amateur-educational sphere, closely linked to academic tradition. Third, the National Shakaijin Cup was incorporated, connecting adult regional amateur football with national competitive structures. Finally, we analysed football events embedded within traditional local festivals, directly reflecting the link between sport and community-based culture.

Given that the research addresses a complex and evolving phenomenon, we employ a multiple case study methodology, grounded in a systematic review of documentary sources. These include recent academic literature on sport and culture in Japan, official reports from Japanese sporting institutions such as the Japan Football Association (JFA) and the J.League, and specialised press articles that capture relevant developments in sports policy and community engagement (44). Documents were selected using keyword-based searches (e.g., “community sports”, “football and culture”, “J.League hometown”), prioritising sources published between 1993 and 2024. We cross-checked sources with institutional repositories and archives to ensure reliability.

To enhance the validity and reliability of our findings, we reached saturation when no new governance patterns or ownership configurations emerged from the four selected cases. Moreover, we clarify that triangulation was achieved through the combination of different data sources (e.g., academic literature, institutional reports, and press articles) and cross-case comparison across events with distinct governance models, allowing for validation and convergence of findings.

The data collected includes academic secondary sources and official documents from Japanese sports organisations, such as annual reports, policy briefs, and community strategy papers, alongside recent literature on local and regional sports development and specialised media coverage (see Appendix A). In this regard, we gave priority to documents published from the establishment of the J.League to the present, with particular attention to studies on local strategies such as the “hometown” policies implemented by the Japanese professional league (40, 45).

This systematic collection was complemented by a specific analytical framework, aimed at identifying patterns related to key variables such as the predominant governance approach (bottom-up or top-down), the nature and type of ownership (corporate, community-based, or hybrid), the cultural elements present in each case, and the specific mechanisms used for the incorporation of cultural expressions, symbolic practices and forms of community participation. Table 2 summarises these dynamics by mapping the main stakeholders involved in Japanese football events, the types of resources they contribute, and their corresponding ownership profiles.

**Table 2:** OL configurations and bottom-up dynamics in Japanese football.

Key stakeholders	Resources contributed	Predominant ownership style
Corporate sponsors	Financial capital, infrastructure, brand visibility	Top-down
Sports federations (JFA, JUFA)	Rules, refereeing, institutional prestige, logistical support	Moderated top-down
Municipal governments	Sports infrastructure, public subsidies, local promotion	Hybrid (local top-down)
Universities and local associations	Volunteers, academic training, on-campus facilities, educational tradition	Bottom-up
Amateur/regional clubs	Local talent, community pride, regional identity, grassroots mobilisation	Bottom-up
Fans and local communities	Social capital, collective identity, popular support, active participation in the events	Bottom-up

Source: own elaboration.

Using this analytic framework, the paper explores how OL has evolved from strictly corporate models, where large industrial companies such as Mitsubishi Motors, Toyota, or Nissan directly managed clubs under a distinctly vertical or top-down logic, towards more participatory hybrid schemes. In our sample of events context, the strategic introduction of professional football marked a fundamental shift, as clubs were explicitly required to establish ties with specific cities under the “local club” or *hometown club* model (46). It represented a significant opening toward local actors such as municipal governments, regional small businesses, and community supporter groups, partially shifting ownership toward a more diversified, polycentric model (7, 47). Emblematic clubs such as Kashima Antlers and Urawa Reds exemplify this transition, evolving from purely corporate entities to hybrid community-based models.

This strategic shift toward a hybrid model (see Table 3) facilitated the achievement of key objectives such as football's territorial expansion, its nationwide popularisation, and, most importantly, strengthening the cultural legitimacy of the events by rooting them in community practices and values (7). This approach has triggered considerable social mobilisation, significantly increased average match attendance and established football as a new local cultural symbol, comparable in significance to traditional Japanese baseball (47–49).

**Table 3:** Evolution of ownership models.

Historical period	Predominant model	Key actors	Examples	Impact on intangible heritage
Pre-1993	Corporate (Top-down)	Industrial conglomerates (*Keiretsu*)	Mitsubishi Motors, Nissan, Toyota	Low
1993–1999 (early J.League years)	Transitional mix (top-down shifting to community-based)	Founding companies, municipalities	Kashima Antlers, Urawa Reds	Moderate, emerging
Post-1999 (*100 Year Plan* era)	Hybrid model (Bottom-up/Top-down)	Local clubs, municipalities, communities	Kashima Antlers, Albirex Niigata	High, sustained

Source: own elaboration.

At the same time, this paper explicitly acknowledges that the new model does not entail the disappearance of traditional corporate sponsorship, but rather its effective integration into hybrid structures that combine strategic elements characteristic of top-down approaches, such as funding, professional infrastructure, and corporate management, with bottom-up mechanisms, as local identity, active fan participation, and community development.

To complement the qualitative case studies and deepen the understanding of how resource configurations affect performance sustainability, we conducted a Data Envelopment Analysis (50, 51) based on club-level data across multiple seasons (2019–2021). DEA is a non-parametric technique used to evaluate the relative efficiency of decision-making units (DMUs), in this case, Japanese professional football clubs, by assessing how effectively they convert resources into performance outcomes. The data was obtained from club financial statements, the Transfermarkt database, and J.League annual reports. Our model comprises three inputs: operating, sponsorship, and participation revenue; wage costs; and market value, aiming to reflect the financial and organizational resources available to each club. The outputs are sporting results: goals scored (a desirable output) and goals conceded (a non-desirable output). We used an input-oriented DEA model with both CRS and VRS assumptions, using the MATLAB R2024b software. All variables were normalised to avoid scale bias, and sensitivity analysis was conducted to test robustness.

The model evaluates technical efficiency under constant and variable returns to scale (CRS, VRS), scale efficiency (SE) in line with previous applications of DEA in sport management research (52–54). This indicates whether a club is operating at an optimal size. In this context, SE helps identify whether performance improvements could stem from adjusting the scale of operations (55). While this analysis does not directly measure OL, it provides indirect evidence of how different governance and resource structures translate into organisational sustainability. Moreover, clubs that operate efficiently tend to integrate community involvement and uphold cultural traditions in their organizational practices (56, 102).

To justify the selection of inputs and outputs, we followed established criteria in sport efficiency studies (57, 58) In this regard, to assess how effectively clubs convert financial and organisational resources into competitive outcomes, thereby indirectly shedding light on the sustainability of different OL configurations, we opted for the following variable selection. The inputs (operating, sponsorship, and participation revenue; wage costs; and market value) were selected to capture the clubs' financial capacity, resource mobilisation, and organisational value. The outputs, goals scored (desirable) and goals conceded (undesirable), were chosen as reliable proxies for sporting performance (103), widely used in DEA applications in football.

DEA provides valuable information on how clubs convert resources into sporting outputs. However, it does not capture enough the symbolic value, emotional ties, or the transmission of cultural practices, dimensions that are central to this study. Therefore, we interpret DEA results in conjunction with qualitative data on stakeholder configurations, community participation, cultural embeddedness, and other relevant factors. The observed alignment between high efficiency scores and strong local engagement in football clubs supports our conceptual claim that diversified and community-anchored OLs promote both organisational and cultural sustainability. Nevertheless, we acknowledge that DEA remains limited in its capacity to assess cultural outcomes directly, and thus serves as a complementary, rather than conclusive, tool in this analysis.

## 4 Results

### 4.1 Professional leagues and the hybrid OL model

In Japanese professional football, OL has had a direct impact on the leagues and their associated events. The J.League, as the top-tier competition, was founded on a hybrid model: a centrally coordinated strategic direction combined with community-based clubs. This model has proven to be sustainable, prosperous and competitive in the context of mid-sized contemporary football leagues. Today, the league comprises 60 clubs across three professional divisions, representing 36 of Japan's 47 prefectures. This expansion is attributed mainly to the policy of promoting football in new regions under the framework of the J League 100 Year Plan (59).

Each club is closely tied to its respective city through formal agreements with local governments and through community-oriented activities, which help cultivate strong popular support. In terms of resources, the league provides a stable overarching framework, in which we can find a primary sponsor, broadcasting contracts, and revenue-sharing mechanisms, among others (45). This implies securing financial and reputational capital at the national and global levels. Meanwhile, clubs mobilize local resources like municipal stadiums, volunteers, small-scale sponsors, and supporter traditions rooted in local culture (7).

In 2020, despite the challenges posed by the pandemic, no club collapsed. Many clubs activated their community networks to secure donations or support, while the central league authority adjusted the calendar and financial structure (60). The top-down/bottom-up collaboration became especially evident as the J League reported that in 2020 alone, clubs carried out over 15,000 local support activities during the pandemic, ranging from educational television programs to meal deliveries, demonstrating the emotional bond with supporters and local communities despite attendance restrictions (61).

Building on this resilience, the OL configuration adopted by the J League offers a compelling lens to assess how structural governance models shape organisational performance. Beyond anecdotal success stories, a closer look at club-level efficiency data reveals clear patterns: the type of OL affects management capacity, as seen during the pandemic, and could correlate with long-term sustainability and social outcomes. Table 4 draws on performance indicators to illustrate how community-based, hybrid, and corporate OLs translate into different efficiency and productivity profiles. Thus, we aim to operationalise the conceptual argument that ownership diversity enhances the league's adaptive capacity (20) and cultural embeddedness.

**Table 4:** Ownership landscape typologies and club efficiency in the J.League (2021).

Club	Estimated OL type	SE (2021)	Competitiveness (2021)	Comment
Albirex Niigata	Community-based	0.851	1.62	High relative efficiency with low economic input. Consolidated local OL
Ventforet Kofu	Hybrid	0.867	1.90	Strong efficiency under a mixed structure. Increasing sustainability
Giravanz Kitakyushu	Hybrid	0.668	0.83	Modest but efficient performance. Notable regional support
Vissel Kobe	Corporate	0.736	1.92	High investment, low efficiency. Corporate OL appears less sustainable
Yokohama F. Marinos	Corporate	0.029	2.08	Maximum PPP but very low efficiency. Performance not easily replicable
Thespakusatsu Gunma	Emerging	0.673	0.98	Modest efficiency with potential for structural improvement
Fujieda MYFC	Emerging	0.914	1.14	High relative efficiency despite small scale
Roasso Kumamoto	Hybrid	0.991	1.93	High performance with notable technical efficiency

Source: own calculations.

Note: Competitiveness refers to average points per game (PPP) obtained by each club during the 2021 season, used here as a proxy indicator of competitive performance on the field, independent of budget or input size (62).

Particularly, clubs with diversified or community-anchored OLs tend to exhibit high efficiency levels despite lower economic inputs, aligning with the RBV logic that intangible and culturally embedded resources can substitute for capital-based strategies. For instance, Albirex Niigata, with a community-based OL, achieved a strong SE score of 0.851 and a competitiveness rating of 1.62. Similarly, Ventforet Kofu, a hybrid club, registered 0.867 SE and 1.90 PPP. In contrast, corporately structured clubs may present overinvestment with suboptimal output conversion. Vissel Kobe (SE = 0.736, PPP = 1.92) and Yokohama F. Marinos (SE = 0.029, PPP = 2.08) exemplify this imbalance: while they achieve strong sporting results, the resource-to-output ratio is poor, suggesting overinvestment and low replicability of their model. These results could empirically suggest the argument that OL diversity and hybrid governance foster cultural, economic, and strategic sustainability.

Beyond organisational performance, results exhibit how specific OL configurations contribute to preserving the ICH surrounding football. Clubs such as Albirex Niigata and Ventforet Kofu, which combine high efficiency with deep local engagement, also serve as anchors for social practices, rituals, and affective bonds that transform their matches into community events rich in cultural meaning. In this context, efficiency should not be understood merely as the optimal conversion of inputs, but rather as an organisational capacity for sustainability that supports the continuity of local traditions, fosters civic participation, and strengthens collective identity through sport (63, 64). Moreover, sustained efficiency among territorially rooted clubs can be interpreted as a proxy indicator of success in preserving culturally valuable events, as efficient clubs often align their operations with community engagement and cultural continuity (56, 65).

### 4.2 All Japan university football championship

This championship, commonly known as the Intercollege, is the annual tournament that brings together Japan's top university teams. Its governance falls under the Japan University Football Association (JUFA), a body composed of representatives from regional university leagues (66). Thus, it is a relatively community-based model within the academic sphere. Although the Japan Football Association (JFA) provides logistical support (e.g., by offering stadiums or referees), the tournament remains autonomous mainly from centralized sport authorities. It represents a case study in bottom-up strategies within a semi-autonomous setting as governance decisions originate primarily from within the network of universities.

The OL of the Intercollege tournament (Table 5) exemplifies how a well-configured OL, grounded in bottom-up dynamics and academic community participation, can function as a strategic intangible resource for both organisational and cultural sustainability. The tournament's architecture, led by JUFA and supported by universities, local authorities, and regional federations, rallies a constellation of actors whose collective contributions reflect the essence of VRIN-based resources. Far from being a top-down commercial event, the Intercollege operates through decentralised decision-making, enabling a strong alignment with local identities and symbolic traditions beyond mere competition.

**Table 5:** Main actors involved in the OL's intercollege tournament.

Actor	Type of actor	Governance role	Resources contributed
JUFA	Academic federation	Overall organisation (format, calendar, venues)	Legitimacy, expertise, university network
Universities	Educational institutions	Direct participation and funding	Volunteers, team budgets, athletes, and institutional identity
JFA	National football federation	Institutional support	Referees, stadiums, visibility
Local communities	Local governments/civil society	Co-organisation of regional rounds	Infrastructure, cultural engagement
MCC Sports (sponsor)	Private company	Title sponsor (since 2021)	Branding, financial input
Regional University Football Federations	Regional Federation	Organisation and operational management	Organisation, venue assignment, cultural-symbolic capital

Source: own elaboration.

The tournament is funded by title sponsorships (e.g., MCC Sports since 2021), JFA support, ticket revenue, and university contributions. The intangible prestige of sports success justifies the transportation, equipment, and logistics expenses that each institution incurs. Thus, the sustainability of these events relies on a diverse organization of labor (OL) involving JUFA, the universities, the host community, and especially the JFA, all of whom have vested interests in upholding the tournament tradition. The multiple-actor arrangement enabled the tournament to continue in 2020 with strict pandemic protocols, demonstrating its resiliency as an institution.

Historical heritage and ICH are closely linked, as both involve the transmission of cultural identity across generations (67). In this regard, the tournament has a long-standing tradition and has served as a breeding ground for numerous professional players since its inaugural edition in 1953. Thus, beyond its sporting function, the tournament represents a form of ICH, as defined by UNESCO (3), insofar as it embodies living traditions transmitted across generations through a community of practice. Moreover, numerous J League clubs recruit standout graduates from the university championship each year. Internationally renowned Japanese stars such as Hidetoshi Nakata and Yasuhito Endo rose to prominence through university football before achieving wider fame. Studies by Guo et al. (68) and Khalil et al. (69) highlight that talent development plays an important role in cultivating cultural transmitters and integrating ICH into professional talent pipelines.

In contrast to international models such as the NCAA in the United States, the Japanese university football system is characterized by its emphasis on community-driven values, and the symbolic transmission of cultural heritage, rather than commercial imperatives. Whereas the NCAA operates within a highly professionalized and revenue-oriented framework, marked by substantial media contracts and centralized institutional control, the All Japan University Football Championship prioritizes an amateur ethos and the preservation of cultural legacy.

Historic rivalries (see Table 6) are also integral to the university football folklore. The Intercollege tournament is traditionally held between late December and early January, typically at neutral venues that rotate across different regions. While not directly addressed in specific ICH literature, the concept aligns with studies like those of Lei (104), which emphasize that ICH is closely tied to cultural memory and communal experiences (70, 71). Likewise, iconic venues and stadiums, as ICH promoters (72, 73), such as Kashima or Toyota have been used in some editions to foster the tournament's prestige. This rotational model also allows various cities to host the competition, allowing local communities to engage in a nationally relevant sporting event. Additionally, there is a broad geographic representation from all regions of Japan, with 28 teams qualifying for the tournament (as of 2024; previously 24).

**Table 6:** Forms of ICH preserved and transmitted through the structure and rituals of the university football tournament.

ICH element	Expression within the tournament
Historic rivalries	Waseda–Keio derby, echoes of Oxbridge traditions
Senpai–Kohai culture	Seniors mentor juniors on and off the pitch
Musical-supporter traditions	University brass bands and cheerleaders from baseball
Fair play as a cultural value	Ritual greetings, venue appreciation, JUFA protocol
Institutional pride and identity	Alumni presence and student chanting in the stands
Dual academic-athletic pathway	Players remain enrolled in degree programmes

Source: own elaboration.

The supporters in the stands, typically students or alumns with some representation from brass bands and cheerleaders, generated a sense of school tradition from collegiate baseball that derived an exceptional student culture. Moreover, traditions, such as the senpai–kohai hierarchies that represented generational ties, are also distinctly visible and reflect the fish-netting of the tournament into the institutional rhythms of campus life in Japan.

### 4.3 The Shakaijin Cup: regional identity and amateur ethos

The Zenkoku Shakaijin Soccer Taikai is an annual tournament that brings together adult amateur (non-university) football clubs across Japan. Shakaijin, meaning “members of society” or “working adults”, indicates its traditional orientation toward company teams and regional clubs outside the school and university circuits. The competition acts as a showcase for regional identity, with participating teams representing champions of each regional league (e.g., Hokkaidō, Tōhoku, Kantō), often with names that highlight local origin (e.g., ReinMeer Aomori, Briobecca Urayasu, Ococias Kyoto).

Moreover, it forms part of the promotion pathway toward higher-tier football, as top-performing teams qualify for the subsequent play-off tournament leading to the Japan Football League (JFL). Thus, the Shakaijin Cup is a key mechanism in the renewal of the Japanese football pyramid, as it has served as a launching pad for numerous clubs currently in J2 and J3, such as Blaublitz Akita, FC Imabari, and Kagoshima United, who began to stand out thanks to their performances in this tournament. According to Wen (105), institutions play a vital role in sustaining and formalizing intangible traditions through structured systems and talent pipelines.

The event follows a concentrated format held over a few days at a rotating host city, bringing all qualifying teams together in one location. This structure turns the tournament into a regional celebration of sport, allowing residents and volunteers to participate. The strong connection between clubs and their home regions is also visible in player composition: many Shakaijin players are locally born, unlike in the J.League, where cross-regional transfers are more common. These clubs, therefore, symbolise retained local talent and act as cultural ambassadors for their prefectures. Notable examples include Honda FC, a corporate amateur team from Hamamatsu, Shizuoka.

The Shakaijin Cup operates under a multi-actor governance model (see Table 7), with responsibilities distributed across institutional and territorial levels. While the JFA plays a mainly symbolic and regulatory role (providing referees, the trophy, and limited subsidies), regional federations and host municipalities handle most operational duties. Regional Football Associations manage team selection and ensure continuity between local leagues and national representation. Furthermore, the annually rotating host city supplies logistical infrastructure, promotes civic engagement, and leverages the event for tourism. In this regard, clubs contribute with players, coaches, and partial funding, often supported by municipal aid and local sponsors. Volunteers and citizens assist with logistics and community outreach, ensuring smooth execution. Functionally, the event's OL rests with regional associations, and its integration into local development agendas further elevates the cultural relevance. In this regard, prefectures often align the Cup with regional identity-building efforts, incorporating cultural activities, such as culinary and folk exchanges (particularly in editions held in Kyūshū).

**Table 7:** OL of the Shakaijin Cup as a semi-professional tournament.

Actor	Type	Governance role	Resources contributed
Japan Football Association (JFA)	National federation	Institutional oversight, basic regulation, and symbolic legitimation	Referees, trophy, minimal subsidies, national promotion
Regional Football Associations	Regional federations	Selection of representative teams, coordination with host cities	League organisation, team nomination, logistical support
Host Municipality (rotating)	Local government	Provision of venues and community coordination	Stadiums, local volunteers, tourism promotion, hospitality
Participating Clubs	Semi-pro/amateur clubs	Direct event participation and community mobilisation	Players, staff, funding (from companies, municipalities), fan engagement
Local Sponsors/employers	Small businesses	Club-level financial and social support	Equipment, transport, time-off for players (many are employees)
Volunteers and citizens	Local civil society	Event execution, cultural support	Match-day assistance, social media, identity-building

Source: own elaboration.

Table 8 synthesises how the Shakaijin Cup functions as a platform for activating and transmitting various elements of ICH. Regional pride is visibly embedded through club names tied to localities and strong community support, reinforcing territorial identity. The amateur ethos is central, with many players requesting time off work to participate.

**Table 8:** Forms of ICH expressed through the Shakaijin Cup.

ICH element	Manifestation in the tournament	Socio-cultural function	Theoretical relationship
Regional pride and representation	Teams named after cities/prefectures; community support for local clubs	Strengthens territorial identity and sense of belonging	(47, 74)
Amateur ethos	Players often request time off work to participate	Embodies effort, humility, and community recognition	(75)
Community engagement	Rotating hosts involve residents, volunteers, and cultural exchange	Temporarily transfers symbolic ownership of the event to the host	(7)
Cultural rituals and fair play	Ceremonial gestures, respect between teams, grassroots camaraderie	Preserves values of Japanese sporting culture	(76)
Interregional exchange	Clubs from all over Japan meet and interact in one location	Builds national cohesion through football	(77)
Institutional resilience	Clubs that remain amateur despite success (e.g., Honda FC)	Symbol of sustainability without financial dependence	(40)
Festivalisation of the event	Integration with regional festivities and tourism promotion	Links football with local customs and regional visibility	(78)

Source: own elaboration.

Community participation is encouraged by the tournament's rotating venue model, which temporarily transfers symbolic ownership of the event to local actors. Furthermore, rituals linked to fair play, such as formal gestures of respect between teams, reinforce traditional values associated with Japanese sporting culture. By bringing together clubs from different regions in a single location, the event also acts as a platform for interregional exchange, contributing to building national cohesion through sporting means. At the same time, the tournament's integration into local festivities and tourism strategies demonstrates a process of festivalization in which football is intertwined with regional customs, strengthening cultural visibility and social participation levels.

The Shakaijin Cup also plays a role in renewing Japan's football pyramid. Numerous clubs competing in J3 and J2 (such as Blaublitz Akita, FC Imabari, or Kagoshima United) first gained national attention by winning or excelling in this tournament before becoming professional. This confirms the event's function as a platform for identifying and promoting successful grassroots projects related to the preservation and promotion of ICH. At the same time, the competition sustains visibility and motivation for amateur clubs that choose not to professionalise, offering them a national title as a concrete seasonal goal.

Overall, the competition thus serves a dual purpose: on the one hand, it acts as a springboard toward professionalization; on the other, it offers a highly symbolic goal for those clubs that choose to remain in the amateur sphere, allowing them to win a prestigious national title.

### 4.4 Local festivals and football

Researchers have documented cases in various regions of Japan in which football is actively integrated into traditional festivities (7, 79, 80). In this regard, these experiences combine inherited cultural practices, such as parades, dances, and religious rituals. A representative example is in municipalities in Shizuoka Prefecture, where children's 7-a-side football tournaments are held during summer festivals in public spaces, accompanied by traditional elements such as food stalls, taiko performances, and evening lantern ceremonies. This setup encourages intergenerational contact with local cultural practices and expands ICH local significance (81).

Similar cases have been observed in rural areas of the Tōhoku region, where interschool competitions are incorporated into the municipal festival calendar. High school sports tournaments are held in these localities with traditional dances such as shishi-odori and closed with cultural events, including fireworks. Thus, these practices transform school events into broader community celebrations and imbue them with cultural significance beyond football.

Researchers can also identify initiatives professional clubs promote in collaboration with local governments, such as the “Football Days” organised by Albirex Niigata during Tanabata Matsuri. On these occasions, children's tournaments are held with festival-themed decorations, including writing wishes on paper strips, fully integrating sports into the traditional ritual. These activities are also promoted as tourism products thanks to the collaboration between sports stakeholders and guardians of tradition. Thus, the organisational structure of these events reflects a form of shared symbolic ownership, where neighbourhood associations and local clubs act as co-producers of the event, with the municipal government as facilitator.

The ICH impacts observed in these integrated sports festivals are multiple. First, they contribute to cultural preservation by reactivating traditions with youth participation and engaging new audiences in heritage practices. Second, they strengthen social cohesion by creating intergenerational meeting spaces. Third, they generate added value for the development of grassroots sports by offering accessible, festive, and visible environments for sports practice, where informal scouting activities by coaches have even been reported. Finally, they foster local economic dynamism by attracting visitors, stimulating trade, and promoting sports and local traditions externally.

Finally, Table 9 cross-sectional comparison allows us to visualize how different football events in Japan operate under distinct governance models (top-down, bottom-up, or hybrid) that activate diverse actors and resources. While the J.League represents a consolidated hybrid model, combining centralized strategic direction with strong community roots, university championships and the Shakaijin Cup illustrate eminently participatory forms of governance, where universities, amateur clubs, and regional associations configure decentralized organizational structures sustained by social capital and institutional commitment. Meanwhile, sports festivals integrated into local holiday calendars reflect a hybrid bottom-up articulation where football is intertwined with cultural practices and tourism as a vector of social cohesion and territorial visibility.

**Table 9:** Japanese football ownership landscape.

Type of event	Dominant governance approach (top-down/bottom-up/hybrid)	Main stakeholders	Key resources contributed
J.League	Hybrid	Corporations, local governments, fans	Financial capital, infrastructure, social capital
University Football Championship	Bottom-up	Universities, JUFA	Academic talent, volunteers, local identity, academic tradition
Shakaijin Cup	Bottom-up with moderate institutional support	Regional teams, local associations, JFA	Local infrastructure, regional talent, social cohesion, regional sponsors
Local Sports Festivals	Hybrid (predominantly bottom-up with minimal top-down support)	Local committees, sports clubs, municipal governments	Community rituals, local volunteers, municipal facilities, tourism promotion

Source: own elaboration.

## 5 Discussion

The main objective of this study was to explore how bottom-up and top-down governance strategies in Japanese football events, within the managerial conceptual framework of the Ownership Landscape (OL) from the Resource-Based View (RBV), contribute to the preservation of ICH. Based on the analysis of four emblematic cases in Japan, the aim was to define and operationalise OL within the RBV framework, evaluate the interactions between bottom-up and top-down governance models, and understand how these configurations facilitate the preservation of intangible heritage.

The results show that events with hybrid or participatory OL tend to generate strategic resources that meet the VRIN criteria (16, 20), thus contributing to their cultural and organizational sustained competitive advantage. For example, the J.League case demonstrated how a hybrid strategy enabled the mobilization of community and corporate resources to ensure effective resilience in the face of crises, as observed during the COVID-19 pandemic. This idea aligns with previous studies on organisational sustainability and intangible resources (82). More specifically, clubs such as Albirex Niigata and Ventforet Kofu exemplify this dynamic, demonstrating high operational efficiency through their ability to combine local resources and corporate strategies effectively.

These findings are consistent with Absalyamov (83), who notes that large-scale sporting and cultural events, along with hybrid governance, can positively sustain economic and social development. Similarly, Kiuri and Teller (73) emphasise how the strategic management of sports facilities' architectural and cultural legacy contributes to identity strengthening and collective memory. Thus, previous literature reinforces our results concerning OL, aligning with our observations in Japan. Shakaijin Cup and local festivals integrate football into broader cultural narratives, whether through rotating tournament venues that foster community cohesion or the inclusion of matches in traditional celebrations such as the shishi-odori in Tōhoku or youth tournaments in Shizuoka.

Furthermore, as Pinson (84) suggests, the perception of sporting events as heritage assets depends mainly on the authenticity they project, an authenticity that is built and maintained through symbolic narratives closely linked to local communities. This idea resonates with our findings. When OL is well-configured, the event's authenticity and emotional connection with the territory are also intensified. Along similar lines, Chappelet (72) highlights the strategic value of these events in territorial marketing, as they consolidate local identity and contribute, in the long term, to strengthening the territorial brand.

From a theoretical perspective, this study contributes significantly to recent calls for the development of the RBV framework (85–87). In doing so, this paper expands previous research that applied RBV to sports events, such as Pianese (19). Regarding ICH, our research highlights how sporting events can act as dynamic mechanisms for activating and transmitting local cultural practices, in line with Malchrowicz-Mośko and Poczta (88) and Ziakas and Costa (2). However, it is essential to acknowledge that the sustainability of a participatory OL is not without tensions, particularly when there is a high degree of financial dependence or state intervention, which can impact its authenticity and long-term effectiveness.

A relevant case is the Chinese Super League, where top-down governance and massive state-backed investment initially fuelled rapid growth (89, 90). However, this model later raised concerns about overreliance on public funding, real estate markets, as well as a growing disconnect from grassroots communities. These factors ultimately undermined the league's cultural legitimacy and long-term stability (91, 92). Interestingly, however, grassroots football and basketball initiatives in China, such as the Su Super League in Jiangsu or the Village Super League and “Village BA” tournaments in Guizhou and Zhejiang, have successfully generated widespread cultural resonance and local engagement. These events are organised primarily through municipal and community collaboration and have drawn thousands of spectators and millions of online viewers. This is particularly interesting as they are fueled by strong local identity, informal rivalries, and digital accessibility. Their popularity demonstrates how decentralised, community-led OLs, with bottom-up strategies, can effectively mobilise symbolic capital and forge deep emotional connections with their audiences as apposite with large-scale corporate funding sport clubs. These configurations can meet the VRIN criteria, especially through their cultural rarity and inimitable embeddedness in local traditions.

While this study focuses specifically on the Japanese context, the conceptualisation of OL as a strategic intangible resource holds broader relevance. In this regard, we can observe similar dynamics between governance structures, resource allocation, and cultural legitimacy in other regions, shaped by their unique institutional and historical contexts. In Europe, for instance, some hybrid models combine municipal support with community engagement. We can see this in events such as the Coppa Italia amateur competitions or Germany's local football cups. In Latin America, grassroots football tournaments often rely on informal yet resilient community-led governance mechanisms, which mobilise symbolic capital and local identity to sustain themselves across generations despite limited formal resources. While OL configurations may differ, the RBV lens allows for a comparative understanding of how events generate, protect, and reproduce intangible cultural assets.

In broader theoretical terms, this study offers interesting implications related to emerging concepts such as the Geopolitical Economy of Sport (93) and soft disempowerment (94) in the sporting context. The ability of sporting events to generate cultural influence or soft power (12, 95, 96) through the active and visible promotion of local cultural practices demonstrates how these platforms can significantly contribute to the geopolitical economy of sport. Zhang, Wen and Li (97) support this idea by highlighting how the perceived authenticity of heritage sporting events reinforces tourist satisfaction and loyalty. This ultimately has a positive influence on the international positioning of the destination. In this context, soft disempowerment, understood as the progressive loss of cultural legitimacy derived from strategies disconnected from the community fabric, is also relevant here, as it explains possible cultural failures of excessively corporatised events. A notable example can be seen in some editions of the Formula E series held in host cities without strong motorsport traditions and community engagement. These events struggled to generate local identification and lasting cultural resonance despite initial fanfare.

Beyond their organisational and cultural relevance, the governance strategies examined in this study intersect meaningfully with broader debates on Corporate Social Responsibility (CSR). Several of the practices identified, such as inclusive stakeholder engagement, community involvement, cultural preservation, and the integration of educational objectives, reflect core CSR principles, particularly those grounded in stakeholder theory (98) and the notion of creating shared value (99). From this perspective, researchers may also understand football events governed through diverse and participatory OLs as vehicles for corporate ethics and social responsibility. This reading also aligns with contemporary approaches (100) that advocate for a more integrated view of sports organisations as contributors to societal value, where economic, social, and cultural dimensions are mutually reinforcing.

Ultimately, the practical implications of this study are clear and relevant for those involved in managing sports events and designing public policy. As Malchrowicz-Mośko and Poczta (88) suggest, small-scale sporting events rooted in local heritage can generate tangible benefits in tourism, social cohesion, and community pride. Our findings reinforce this perspective and suggest that a balanced model of governance that combines strategic direction from above with active grassroots participation can enhance the sustainability of organisations while safeguarding ICH. This means acknowledging the strategic value of diversified and well-aligned ownership and governance models for sports entities and institutions to secure their viability and deepen their cultural legitimacy. Thus, when managed thoughtfully, sporting events can evolve into genuine engines of community identity and long-term local development.

## 6 Conclusion

This study has examined how different configurations of the Ownership Landscape (OL), when analyzed through the Resource-Based View (RBV), influence the promotion of Intangible Cultural Heritage (ICH) in sporting events in Japan. We analyze four emblematic football-related cases, ranging from the professionalized J.League to amateur and community-embedded tournaments.

Our findings suggest that events governed through participatory or hybrid OLs are more likely to activate VRIN resources that ensure long-term competitiveness and cultural embeddedness. The J League's ability to integrate national-level sponsorship with local community identity, the Intercollege's academic-based traditions, and the Shakaijin Cup's regional rootedness all demonstrate how sports can serve as living vessels of cultural expression. Moreover, these cases demonstrate that football events reflect existing traditions and also generate new cultural forms, reinforcing collective memory and becoming institutionalized as intangible heritage.

The research also highlights the need to move beyond simplistic top-down or economic impact models when evaluating sporting events. Instead, it advocates for an analytical lens that incorporates cultural dynamics, stakeholder diversity, and the symbolic functions of sport. In doing so, this paper advances the theoretical development of the RBV by extending it to culturally embedded, multi-actor organisational ecosystems.

Ultimately, this study provides practical guidance for sports managers, policymakers, and cultural planners: sustainable event governance should not prioritize financial returns or visibility solely but must also incorporate inclusive ownership models that foster local engagement and authenticity. When aligned with the social fabric of their territories, sporting events become more than competitions as they emerge as mechanisms for intergenerational cultural transmission and vehicles for community development. Thus, this research proposes a set of adaptable principles to support the culturally sensitive governance of sports events, offering policymakers and practitioners with a flexible framework they can draw on to design governance models that balance high performance with the safeguarding of ICH. These principles include encouraging hybrid ownership structures that combine strategic leadership with grassroots involvement; weaving cultural narratives into the identity of events to strengthen their authenticity; and bringing in a broad range of stakeholders to ensure that organisational sustainability goes hand in hand with community trust and relevance.

### 6.1 Limitations and future research

One key limitation of this study lies in the absence of a standardized metric to quantify the OL. While we have attempted to approximate OL's organizational effects through efficiency scores, using DEA indicators as a proxy, and acknowledge previous efforts that employed Herfindahl indices to measure economic concentration in club ownership structures, OL remains primarily conceptual in this paper. This constrains the ability to conduct statistical comparisons or hypothesis testing. Additionally, the study's cultural and institutional focus on Japan may limit the transferability of its conclusions to other contexts with different governance traditions.

Future research should build on these initial proxy efforts by developing a robust, multidimensional index to quantify OL more systematically. This could integrate stakeholder diversity, ownership dispersion, community involvement, and governance type. Moreover, conceptualizing OL as a key facilitator of sustained competitive advantage and as a promoter of ICH opens new avenues for understanding how the strategic management of intangible resources can optimize cultural preservation within sports. Finally, future research could expand this framework by applying it across diverse cultural settings to refine its explanatory power and identify context-specific variables that influence its effectiveness.

## Data Availability

The original contributions presented in the study are included in the article/Supplementary Material, further inquiries can be directed to the corresponding authors.

## Appendix

Appendix A Documentary source overview table.

**Table:**

Source type	Number	Date range	Source examples	Type/justification
Peer-reviewed academic literature	41	1993–2024	Sugiyama et al. (9), Dolles and Söderman (40), Nogawa and Maeda (48)	Empirical grounding on Japanese football, sport and heritage
JFA official reports and materials	42	1993–2024	JFA grassroots declaration, white paper, management guides	National sport governance and heritage policies
J.League documents and strategies	18	1999–2024	J League annual reports, financial reports, hometown strategies	Institutional evolution, hybrid models, community-based OL
Governmental and municipal documents	6	2016–2024	Niigata City (2020). Niigata City Sports Promotion Plan. https://www.city.niigata.lg.jp/	Academic-sporting intersection, university governance
News media (NHK, Asahi, The Nippon Foundation…)	12	2010–2024	The Nippon Foundation (45). J. League Sustainability Activation Project. https://en.nippon-foundation.or.jp/	Coverage of social dynamics, club crises, community action

Source: own elaboration.
